# Generalization Gradients in Cued and Contextual Pain-Related Fear: An Experimental Study in Healthy Participants

**DOI:** 10.3389/fnhum.2013.00345

**Published:** 2013-07-05

**Authors:** Ann Meulders, Nele Vandebroek, Bram Vervliet, Johan W. S. Vlaeyen

**Affiliations:** ^1^Department of Psychology, University of Leuven, Leuven, Belgium; ^2^Center of Excellence Generalization Research in Ill Health and Psychopathology, University of Leuven, Leuven, Belgium; ^3^Department of Clinical Psychological Science, Maastricht University, Maastricht, Netherlands

**Keywords:** fear conditioning, fear generalization, unpredictability, contextual pain-related fear, cued pain-related fear, generalization gradient

## Abstract

Increasing evidence supports the notion that pain-related fear plays a key role in the transition from acute to chronic pain. Recent experimental data show that associative learning processes are involved in the acquisition of pain-related fear. An intriguing yet underinvestigated question entails how spreading of pain-related fear in chronic pain occurs. In a voluntary movement paradigm in which one arm movement (CS+) was followed by a painful stimulus and another was not (CS−) in the predictable group and painful stimuli were delivered during the intertrial interval (context alone) in the unpredictable group, we tested generalization of fear to six novel generalization movements (GSs) with varying levels of similarity between the original CS+ movement and CS− movement. Healthy participants (*N* = 58) were randomly assigned to the predictable or unpredictable group. Fear was measured via verbal ratings and eyeblink startle responses. Results indicated that cued pain-related fear spreads selectively to novel movements that are proprioceptively more similar to the CS+ than to those similar to the CS− in the predictable group, but not in the unpredictable group. This is the first study to demonstrate a generalization gradient of cued pain-related fear. However, this effect was only present in the startle eyeblink responses, but not in the verbal ratings. Taken together, this paradigm represents a novel tool to scrutinize the largely understudied phenomenon of the spreading of fear and avoidance in patients with chronic musculoskeletal pain and mapping possible pathological differences in generalization gradients and the spreading of pain in patients as compared with healthy controls.

## Introduction

It is commonly recognized that the relationship between nociceptive input and suffering in chronic pain is not straightforward (Fordyce, 1988). Evidence suggests that a great part of the patients’ suffering is not due to the pain itself, but rather to the exaggerated emotional responses and the broad range of escape/avoidance behaviors accompanying it (Fordyce, 1988; Crombez et al., 1999). Consistent with this view, prevailing theoretical models consider chronic musculoskeletal pain disability in terms of the strength of fear of movement/(re)injury. Current fear-avoidance models indeed suggest that high *fear of movement-related pain* is an underlying deviant pathology that motivates patients to disrupt their daily activities instigating a vicious circle of pain, avoidance, hypervigilance, depression, and disuse. In comparison, patients with low fear of movement-related pain remain active after an acute pain episode, which in turn leads to functional recovery (Vlaeyen and Linton, 2000, 2012; Leeuw et al., 2007a).

According to fear-avoidance models fear of movement/(re)injury is acquired through associative learning processes, that is, initially neutral movements/activities (conditioned stimuli, CSs) that are associated with (increases in) pain (unconditioned stimulus, US) start to elicit defensive responses such as fear and avoidance (conditioned responses, CR). In chronic pain patients, fear and avoidance are often not restricted to movements/activities that were associated with pain during the initial pain episode (Leeuw et al., 2007b). Therefore, a fascinating yet empirically underinvestigated question entails how spreading of pain-related fear and avoidance occurs. Associative learning theory predicts that conditioned fear responses extend to a range of novel stimuli resembling the original fear-eliciting CS, that is, more similar generalization stimuli (GSs) activate more similar responses (i.e., *generalization gradient*). Stimulus generalization is a highly adaptive mechanism, because the ability to detect similarities between unique but related stimuli may contribute to avoiding harm in a dynamic environment (Kalish, 1969; Honig and Urcuioli, 1981; Pearce, 1987; Ghirlanda and Enquist, 2003; Lissek et al., 2008). Yet, together with reducing the risk of missing positive threat alarms, generalization bears an increased risk to respond to false threat alarms, which might be the case in persistent fear and avoidance behavior in chronic pain. In line with this reasoning, recent etiological accounts of anxiety disorders suggest that not fear intensity, but fear *(over)generalization* to novel, albeit similar settings is the central pathogenic marker in certain anxiety disorders (Lissek and Grillon, 2010; Lissek et al., 2010). A second mechanism that might contribute to the exacerbation and maintenance of chronic pain and disability is operant conditioning (Skinner, 1953). That is, successful escape or avoidance of situations, movements, and activities that induce fear and possibly pain can be instrumentally reinforced (Fordyce et al., 1973, 1982; Philips, 1987; McCracken and Samuel, 2007). In the same vein, situations and movements/activities that resemble the feared situations and movements/activities might be avoided as well, leading to the spreading of conditioned avoidance behavior.

Another interesting observation in the anxiety literature that is worth further scrutiny with regard to pain-related fear is that fear conditioning is not a steady state phenomenon but a dynamic process varying as a function of the contingencies between the CS and the US (Grillon, 2002, 2008; Grillon et al., 2006). In particular, *cued fear* is observed upon the presentation of a well-defined, short-lasting CS, and this response quickly subsides after the offset of the fear-eliciting CS. In the absence of a clear threat signal however, *contextual fear* gradually develops as static environmental cues act as continuous reminders of the US without signaling the exact time of its occurrence or non-occurrence (i.e., safety periods). Hence, contextual cues entail more unpredictability regarding the exact occurrence of the US *relative* to discrete cues. In the same vein, two types of pain-related fear can be distinguished depending on the temporal (un)predictability of the US: cued and contextual pain-related fear. Cued pain-related fear is experimentally induced by predictable pain (i.e., pairings of movement and pain), whereas contextual pain-related fear is induced by unpredictable pain (i.e., pain explicitly unpaired with movements) (Meulders et al., 2011; Meulders and Vlaeyen, 2012).

A recent study in our lab (Meulders and Vlaeyen, 2013a) investigated stimulus generalization using a voluntary movement conditioning paradigm in which, one arm movement (e.g., moving to the left, CS+) was followed by a painful stimulus and another was not (e.g., moving to the right, CS−) in the predictable condition, whereas pain stimuli were never delivered contingent upon the movements (i.e., moving up and down) but during the intertrial interval (ITI) in the unpredictable condition. In this particular set-up spreading of fearful responding to novel diagonal movements (e.g., left-top, right-top, left-bottom, right-bottom) was tested. These movements (GSs) had only one feature in common with the either the original CS+ or CS−, hence gradients could not be calculated. Therefore, the present study was designed to examine whether stimulus generalization of cued pain-related fear is characterized by a gradient. In order to do so, we used an adapted version of the voluntary movement paradigm using six generalization stimuli (GSs) with varying levels of similarity between the original CS+ and CS−. Healthy participants were randomly assigned to the predictable or unpredictable group. In the predictable group, one arm movement (CS+) was followed by the pain-US and another was not (CS−), but in the unpredictable group, painful stimuli were delivered during the ITI. Fear was measured via verbal ratings and eyeblink startle responses. We hypothesized that: (1) cued pain-related fear spreads selectively to new movements that are more similar to the CS+ than to those similar to the CS− (i.e., fear generalization gradient in the predictable group), (2) generalization gradients are flattened in the unpredictable group compared with the predictable group.

## Materials and Methods

### Participants

Seventy healthy female undergraduate psychology students of the University of Leuven (*M*_age_ = 20.19 years; SD_age_ = 1.66, range = 19–29 years), volunteered to participate in this study as a partial fulfillment of course requirements or in exchange for a monetary compensation of 12. Participants were recruited using the departmental experiment management system (EMS; Sona Systems Ltd.), or by means of advertisements distributed at the University of Leuven. Participants completed a general health checklist to confirm they did not have one or more of the following conditions: cardiovascular disease, neurological disease, musculoskeletal disorder, or other pain-related conditions, psychiatric disorders, cardiac pacemaker, or the presence of any other electronic, medical devices, uncorrected vision/hearing problems, injury on hand/wrist, recent use of analgesic, anxiolytic, or antidepressant medication, being pregnant, being under 18 years old, and being a non-native Dutch/Flemish speaker. All participants provided written informed consent and were told that they could decline their participation at any given moment during the experiment. Eight participants were excluded because they did not meet our inclusion criteria. Of the remaining 62 participants that actually participated in the study, 4 were excluded due to technical difficulties, leaving a total of 58 participants to be included in the statistical data-analysis. Participants were randomly assigned to the predictable pain group (*n* = 29) or the unpredictable pain group (*n* = 29). The study protocol was approved by the ethical committee of the Department of Psychology of Leuven University.

### Software

The experiment was run on a Windows XP computer (Dell OptiPlex 755) with 2 GB RAM and an Intel Core2 Duo processor. The visual stimuli were presented on a 19-inch computer screen. The presentation of the stimuli and the data acquisition was controlled with the free software package Affect (version 4.0) (Spruyt et al., 2010) and the data were stored using a National Instruments data acquisition card.

### Stimulus material

**Figure 1:**
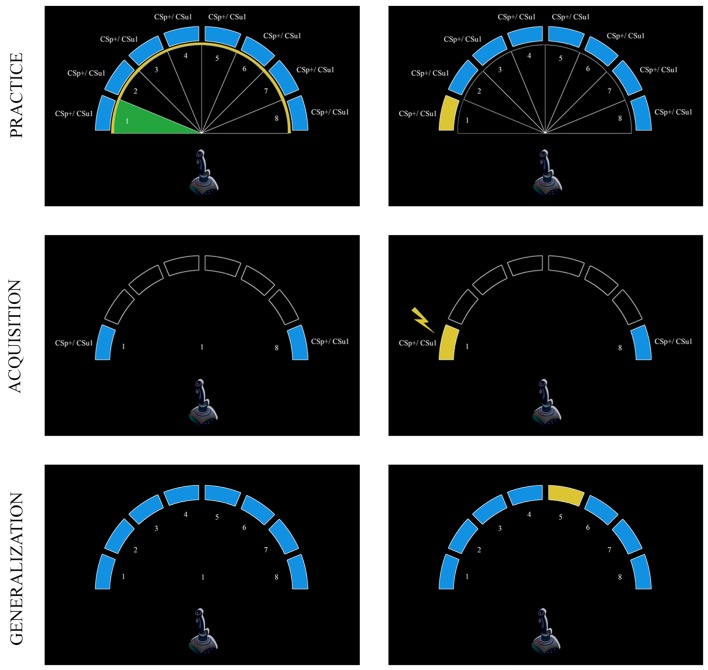
**Schematic overview of the experimental task during the practice, acquisition, and generalization phases**. The eight equally spaced movement quadrants that served as CSs (1 and 8) and GSs (2–7) were delineated by white borders during the practice phase (upper panel). In the predictable group, the CSs and GSs are referred to as CS_p_+, CS_p_−, and GS_p_1–GS_p_6; these stimuli are referred to as CS_u_1, CS_u_2, GS_u_1–GS_u_6 in the unpredictable group. Participants had to move the joystick in the area that colored green and had to aim toward the yellow border. At the end of each of these movement areas a blue bar was positioned; the corresponding blue bar turned yellow when a movement was successfully performed. During the acquisition phase (middle panel), participants only carried out two movements (CSs). After “+” was presented in the middle of the screen, this was replaced by a number that indicated the movement direction (1 or 8). In the predictable group one of these movements was followed by a pain-US (CS_p_+, i.e., left) indicated by a lightning bolt, whereas the other movement (CS_p_−, i.e., right) was never followed by the pain-US. In the unpredictable group, movements (CS_u_1/CS_u_2) were never followed by the pain-US, but it was delivered during the ITI. Finally, during the generalization phase (lower panel) participants had to perform all movements (GSs and CSs) under extinction (i.e., none of the movements were followed by the pain-US).

The CSs and the generalization stimuli (GSs) were eight, equally spaced movement quadrants (Figure 1). These proprioceptive stimuli consisted of moving a (Logitech Attack 3) joystick with the dominant hand within one of the eight movement quadrants. Movements in quadrant 1 (CS_p_+/CS_u_1) and quadrant 8 (CS_p_−/CS_u_2) served as the CSs, and movements in quadrants 2–7 (GS_p_1–6/GS_u_1–6), served as the GSs in both groups. For half of the participants in the predictable group the movement in quadrant 1 (i.e., moving the joystick to the left) was followed by the pain (CS_p_+) and the movement in quadrant 8 (i.e., moving the joystick to the right) was never followed by pain (CS_p_−), whereas for the other half of the participants this combination was reversed. The pain-US was a 50 ms electrocutaneous stimulus, generated by commercial constant current stimulator (DS7 Digitimer, Welwyn Garden City, UK). Pain stimuli were administered to the wrist of the dominant hand through two surface SensorMedics electrodes (8 mm diameter), spaced approximately 1 cm apart, and filled with K-Y gel. Prior to the experiment, the experimental pain-US was individually calibrated. Starting off with an intensity of 1 mA, the stimulus intensity was gradually increased in steps of 1–4 mA until participants rated the stimulus to be “*significantly painful and demanding some effort to tolerate*.” Self-reported pain intensity was rated on an 11-point Likert scale ranging from 0 to 10, with “0” meaning no pain and “10” meaning the worst imaginable pain; a stimulus intensity of 8 on this scale was targeted. The mean self-reported stimulus intensity was 7.69 (SD = 1.16, range 5–10). The mean physical stimulus intensity was 23.78 mA (SD = 15.36, range 6–92 mA). We observed no differences between the predictable and the unpredictable groups with respect to the subjective intensity of the pain-US (predictable group: *M* = 7.66, SD = 1.11, unpredictable group: *M* = 7.72, SD = 0.22), *t*(56) = −0.22, *p* = 0.82, or the stimulus intensity in milliamperes (predictable group: *M* = 24.28 mA, SD = 15.45, unpredictable group: *M* = 23.28 mA, SD = 15.53), *t*(56) = 0.25, *p* = 0.81.

### Outcome measures

#### Eyeblink startle modulation

Orbicularis Oculi Electromyographic (EMG) activity was recorded with three re-usable Ag/AgCl SensorMedics electrodes (4 mm diameter) filled with electrolyte gel. Before attaching the electrodes on the left side of the face according to the site specifications proposed by Blumenthal et al. (2005), the skin was cleaned using an exfoliating peeling cream to reduce inter-electrode resistance. Startle eyeblink reflexes were elicited by a 100 dB burst white noise with instantaneous rise time presented binaurally for 50 ms through headphones (Hoher, stereo headphones, HF92). The raw EMG signal was amplified by a Coulbourn isolated bioamplifier, with band pass filter (LabLinc v75-04) with a cut-off frequency of 13 Hz (low pass filter) and 500 Hz (high pass filter). The signal was rectified online and smoothed by a Coulbourn multifunction integrator (LabLinc v76–23A) with a time constant of 20 ms. The EMG signal was sampled at 1000 Hz from 500 ms before the onset of the auditory startle probe until 1000 ms after probe onset. Eyeblink startle responses elicited by startle probes delivered during the CS/GS movements served as an index of cued pain-related fear and responses elicited by startle probes during the ITI served as an index of contextual pain-related fear.

#### Fear of movement-related pain ratings during the CSs and GSs

After each block during the acquisition and the generalization phase, participants rated the extent to which they were afraid of performing a certain movement on a computerized VAS with anchor points “*not fearful at all*” to “*the worst fear imaginable*.”

### Manipulation checks

#### Retrospective pain-US expectancy

As a manipulation check, pain-US expectancy was assessed at the end of the experiment. Participants indicated for both CSs to which extent they expected to receive a painful stimulus on a computerized VAS with anchors “not at all” and “very much.” The VAS was coded from 0 to 100.

#### Retrospective affective valence of the CSs

After the experiment and as a manipulation check, a computerized version of the Self-Assessment Manikin (SAM) (Bradley and Lang, 1994) consisting of five pictographs, ranging from a smiling, happy figure to a frowning, unhappy figure, was used to measure the affective valence of the CS movements. Participants rated how they felt when performing the respective CSs movements. Scores ranged from 1 “very happy” to 5 “very unhappy.”

### Experimental setting

Participants were seated in a comfortable chair at eye level approximately 60 cm from the computer screen. The sound-attenuated experimental room was located adjacent to the experimenter’s room. Communication with the experimenter was possible through an intercom system; participants and their physiological responses were monitored online by means of a closed-circuit TV installation. Central lightening was turned off but dimmed light was always available in the experimental room.

### Procedure

After obtaining written informed consent, the electrodes for the eyeblink startle measures and the electrocutaneous stimulation were attached. Next, participants went through the four experimental phases described below (see Table 1 for a detailed study design overview), and completed the pain-US expectancy ratings and the SAM scales. After the experiment, participants were thoroughly debriefed.

**Table 1:** **Experimental design**.

Group	Practice phase	Startle habituation	Acquisition phase	Generalization phase
Predictable group	1 × CS_p_+	Nine startle probes (9)	32 × CS_p_+ (8)*	8 × CS_p_+ (4)
	1 × GS_p_1		32 × CS_p_−(8)	8 × GS_p_1 (4)
	1 × GS_p_2		64 × ITI (8)	8 × GS_p_2 (4)
	1 × GS_p_3			8 × GS_p_3 (4)
	1 × GS_p_4			8 × GS_p_4 (4)
	1 × GS_p_5			8 × GS_p_5 (4)
	1 × GS_p_6			8 × GS_p_6 (4)
	1 × CS_p_−			8 × CS_p_− (4)
				64 × ITI (4)
Unpredictable group	1 × CS_u_1	Nine startle probes (9)	32 × CS_u_1 (8)	8 × CS_u_1 (4)
	1 × GS_u_1		32 × CS_u_2 (8)	8 × GS_u_1 (4)
	1 × GS_u_2		64 × ITI (8)	8 × GS_u_2 (4)
	1 × GS_u_3		32 × pain-US (during ITI)*	8 × GS_u_3 (4)
	1 × GS_u_4			8 × GS_u_4 (4)
	1 × GS_u_5			8 × GS_u_5 (4)
	1 × GS_u_6			8 × GS_u_6 (4)
	1 × CS_u_2			8 × CS_u_2 (4)
				64 × ITI (4)

*The number of startle probes given during each movement are placed between brackets. The asterisk indicates that the pain-US was administered. CS, conditioned stimulus; GS, generalization stimulus; ITI, intertrial interval*.

#### Practice phase

Detailed oral and written instructions were provided to the participants before the onset of the experiment to make sure that the purpose of the joystick task was clear. Their main task was to move the joystick toward eight equally sized blue targets that were positioned at the borders of eight movement quadrants (see Figure 1). On each trial, the eight delineated, numbered (1–8) movement quadrants were made visible for the participants by white lines that separated each quadrant from the ones adjacent to it. In this way, participants could identify the valid “movement area” ^1^ covering each quadrant. The borders of the movement quadrants, which participants had to reach in order to hit the target regions were marked with a yellow circle as a means to learn them what constituted a “full” movement. After a 7 s pre-CS ITI, a fixation cross was presented in the middle of the computer screen for 2 s, which subsequently transformed into a randomly selected number ranging from 1 to 8. This number corresponded with one of the numbers depicted in the eight movement quadrants, and informed the participant within which movement quadrant they had to move the joystick during that given trial. The number was presented for 3 s. Participants had to perform the signaled movement as quickly and accurately as possible, and then a 7 s post-CS ITI was inserted before the next trial began. At the beginning of each trial the mouse cursor was positioned in the middle of the screen and the joystick was standing upright and centered, in the resting position. Whenever the mouse cursor representing the movement of the joystick correctly entered the signaled movement area, this area turned green (valid movement), but when the mouse cursor wrongly entered another movement area than the signaled one, this area turned red (invalid movement) and an error message “*incorrect movement, please try again*” appeared in the middle of the screen for 3 s. Whenever the mouse cursor representing the movement of the joystick entered a valid target region, then the blue bar of the corresponding movement quadrant turned yellow, indicating that the movement was performed successfully. To ensure a steady baseline performance, blue bars did not change color in case of an invalid movement. When participants completed eight valid movements (one successful movement within each quadrant) the training phase was aborted and the next phase began. No startle probes or electrocutaneous stimulation was delivered during this phase of the experiment.

#### Startle habituation phase

Nine startle probes were delivered during the habituation phase (ITI = 18–25 s) to circumvent confounds in the data due to large startle responses to the first few probes. Startle habituation trials were excluded from the statistical analysis.

#### Acquisition phase

This phase was similar to the practice phase, except that: (1) the boundaries of the eight movement quadrants and the yellow circle marking the borders of these quadrants were no longer visible. (2) The range of movements in this phase was restricted to quadrants 1 (i.e., movement straight to the left) and 8 (i.e., movement straight to the right), indicated by a blue bar on either side. (3) The movement areas did not turn green or red anymore when the mouse cursor of the joystick reached a valid (green) or invalid (red) movement area. (4) The error message was no longer displayed on the screen when participants reached an invalid target region. (5) Startle probes and pain-USs were presented during this phase.

**Figure 2:**
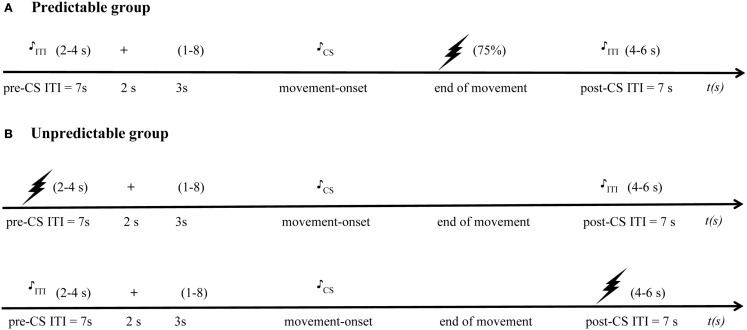
**Detailed trial timing schema**. The drawing of a lightning bolt represents the pain-US, a drawing of a musical note represents the startle probe presentation, and the “+” represents the starting signal, “(1–8)” refers to the presentation of a number between 1 and 8 representing the quadrant in which the participants have to move. **(A)** In the predictable group, during the acquisition phase, pain-USs are delivered in 75% of the trials after the CS+ movements, but not after the CS- movements. On each trial only one startle probe was presented: in the predictable group, this could be during the CS movement or during the pre-CS ITI or post-CS ITI. **(B)** In the unpredictable group pain-USs are delivered at the same rate either during the pre- or post-CS intertrial interval (ITI) and startle probes also could be presented during the CS movement or during the ITI – when the pain-US was presented in the pre-CS ITI, probes were presented in the post-CS ITI, and if the pain-US was presented during the post-CS ITI, probes were delivered during the pre-CS ITI. In both groups, no pain-USs were delivered during the generalization phase.

The acquisition phase consisted of two blocks of 16 trials. Each trial contained two randomly ordered movements, one CS_p_+ movement, and one CS_p_− movement in the predictable group, and one CS_u_1 movement, and one CS_u_2 movement in the unpredictable group. Although the duration of the CS movement itself was of variable length depending on the participants’ response latency and movement speed, there was a fixed ITI consisting of a 7 s pre-CS interval and a 7 s post-CS interval. In the predictable group, a CS_p_+ movement was paired with a painful US in 75% of the trials (12 pain-USs per block), whereas a CS_p_− movement was never followed by the pain-US. In contrast, pain-USs were explicitly unpaired with both the CS_u_1 and CS_u_2 movements in the unpredictable group, and were delivered during the ITI, while keeping the number of pain-USs equal in both groups. In the predictable group, the pain-US was presented immediately after the CS_p_+ movement. In the unpredictable group, the pain-US was delivered in a time window of 2–4 s after the onset of the pre-CS ITI or 4–6 s after onset of the post-CS ITI. A total of 12 startle probes were presented within each acquisition block; eight startle probes were presented during the CSs (four during CS_p_+/CS_u_1 and four during CS_p_−/CS_u_2), and four probes were administered during the ITI (two probes in the 2–4 s time window of the pre-CS ITI, and two probes in the 4–6 s time window of the post-CS ITI) (see Figure 2 for the detailed trial timing). Due to concerns that the startle response during the ITI would be confounded by direct responses to the pain-US in the unpredictable group, startle probes were administered in the pre-CS interval when the pain-US was administered in the post-CS interval, and vice versa. Participants were never verbally informed about the CS-US contingency.

#### Generalization phase

To test the fear of movement-related pain generalization gradient, participants performed unreinforced movements in *each* of the eight quadrants (see Figure 1), while the rest of the joystick task remained the same as during acquisition. The generalization phase consisted of two blocks of four trials. Within one trial, participants had to perform eight movements in randomized order, one movement to each quadrant. The trial flow and timing was similar to that described in the acquisition phase. Startle probes were presented twice for each CS/GS within each block, and twice during the ITI (one probe in the 2–4 s time window of the pre-CS ITI and one in the 4–6 s time window of the post-CS ITI). No pain-USs were delivered during this phase.

### Response definition

The startle data were treated offline with PSPHA, a modular script based program for analyzing psychophysiological data (de Clercq et al., 2006). Each startle waveform was visually inspected for technical abnormalities and artifacts, and considered invalid if the baseline period was contaminated with noise (e.g., movement artifact) or if a spontaneous or voluntary blink occurred during a 1–20 ms time window after probe onset. Based on this response qualification process, 2.10% of the startle responses were identified as being invalid. Afterward, for all valid trials, startle peak amplitude was defined as the maximal EMG value within a response onset window of 21–175 ms after probe onset. If multiple peaks occurred, the maximum value within this time window was still identified as the peak. Every peak response was scored by subtracting its baseline score (= average EMG signal between 1 and 20 ms after probe onset). Raw scores were transformed to *T*-scores to account for inter-individual differences in physiological reactivity and to optimize the visualization of the startle data (i.e., avoid negative values on the *Y*-axis).

### Overview of the statistical data-analysis

Startle responses to every two intermediate GSs were averaged together to form the mean level of responding for that class of movement, thereby reducing the number of levels for the GSs from six to three (class 1: GS1–2; class 2: GS3–4; class 3: GS5–6). The decision to collapse every intermediaries took into account both the concerns that treating each of six GSs as a separate class would require a very long experiment (leading to excessive startle habituation, extinction, and subject fatigue) and that having only three gradients-of-movement would not allow a gradual enough continuum between the CS+ and the CS−. Therefore, we chose to have three classes of intermediaries with two types of movements in each class to evaluate the generalization gradient. That way, each intermediary required only half as many trials (Lissek et al., 2008). A similar procedure was applied to the fear of movement-related pain ratings. Both outcome measures (startle response and verbal fear ratings) were analyzed with separate repeated measures ANOVAs for the acquisition and the generalization phase with as between-subject (BS) factor group (predictable/unpredictable) and as within-subject (WS) factor stimulus type [2 (3) ^2^ levels in the acquisition phase, 5(6) levels in the generalization phase]. We hypothesized a group by quadrant interaction. More specifically, in the acquisition phase we expected the startle response and fear to be elevated in the CS+ than in the CS− quadrant in the predictable group, and we expected no difference between both CS movements in the unpredictable group. Furthermore, we expected the startle responses during the ITI to be higher in the unpredictable group than in the predictable group. In the generalization phase, we expected a group × linear quadrant interaction, that is, a decrease of startle response and verbal fear ratings with increasing distance from the CS+ quadrant in the predictable group, and no relation between quadrant and startle response or self-reported fear in the unpredictable group. Greenhouse–Geisser corrections are reported when applicable, that is, for effects involving a WSs factor with more than two levels. Generalization gradients were further evaluated using Fisher’s LSD multiple comparisons. This test is known to have good power for detecting existing differences. Its vulnerability is generally a family-wise error rate that increases easily, unless the means are assumed to be simply ordered (Nashimoto and Wright, 2005). For all statistical tests, the α-level was set at 0.05.

## Results

### Eyeblink startle modulation

#### Fear acquisition

**Figure 3:**
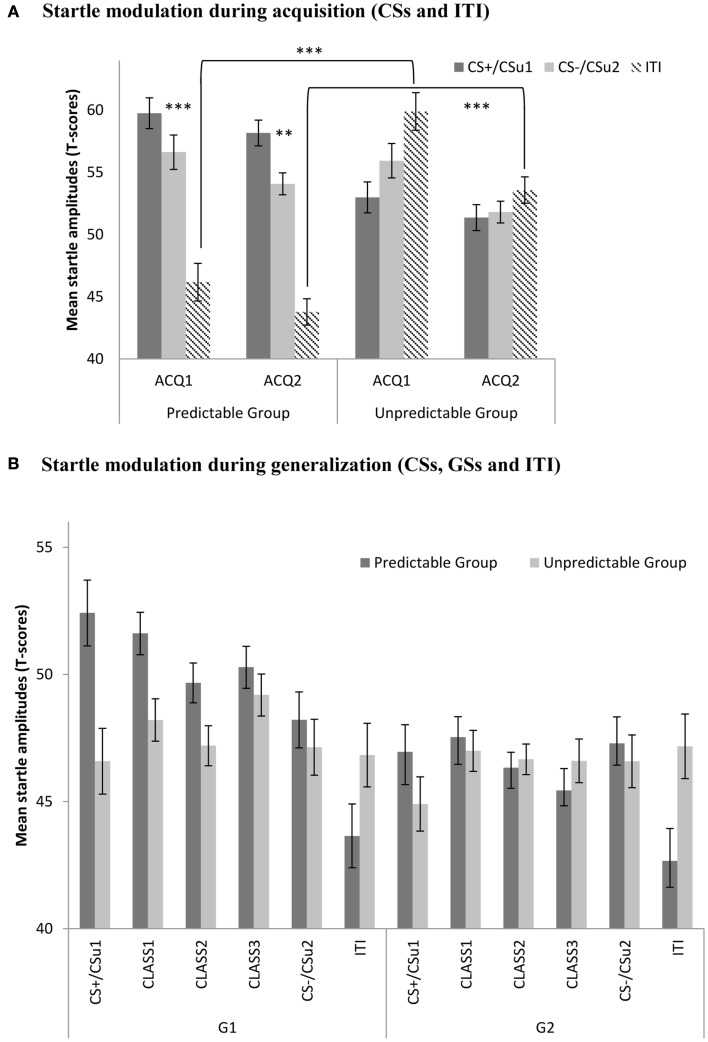
**(A)** Mean eyeblink startle amplitudes (+SE’s) during the CS movements and the ITI in the predictable (CS+/CS−) and the unpredictable group (CS_u_1/CS_u_2) during the two acquisition blocks (ACQ1-2), and **(B)** mean eyeblink startle amplitudes (+SE’s) during the CS movements, the classes of GS movements (CLASS1-3) and the ITI in the predictable and the unpredictable group during the generalization blocks (G1–G2). **(A)** Startle modulation during acquisition (CSs and ITI). **(B)** Startle modulation during generalization (CSs, GSs, and ITI).

Eyeblink startle responses during acquisition were analyzed using a 2 (Group: predictable vs. unpredictable) × 2 (Block: ACQ1 vs. ACQ2) × 3 (Stimulus type; CS+ vs. CS− vs. ITI) mixed (RM) ANOVA. This analysis yielded significant main effects for block, *F*(1, 56) = 16.58, *p* < 0.001, and stimulus type, *F*(1, 56) = 16.69, *p* < 0.001. Interestingly, the stimulus type × group interaction was significant, *F*(1, 56) = 61.37, *p* < 0.001, indicating that startle responses to the different movements differed between the predictable and unpredictable group. As expected, planned comparisons revealed higher startle responses to the CS+ than to the CS− in the predictable group by the end of acquisition (ACQ2), *F*(1, 56) = 10.41, *p* < 0.01, while there was no such difference in the unpredictable group, *F* < 1 (see Figure 3). Further, planned comparisons confirmed that ITI startle responses were significantly elevated in the unpredictable group compared with the predictable group by the end of acquisition (ACQ2), *F*(1, 56) = 42.64, *p* < 0.001.

#### Fear generalization

We used a 2 (Group: predictable vs. unpredictable) × 2 (Block: G1 vs. G2) × 5 (Stimulus type: CS+ vs. class1 vs. class2 vs. class 3 vs. CS−) mixed (RM)ANOVA. This analysis showed main effects for group, *F*(1, 56) = 6.31, *p* < 0.05, and block, *F*(1, 56) = 26.18, *p* < 0.001. Both the block × condition, *F*(1, 56) = 6.00, *p* < 0.05, the stimulus type × block, *F*(4, 224) = 2.76, *p* < 0.05, as well as the stimulus type × condition, *F*(4, 224) = 2.61, *p* < 0.05, interactions were significant. The three-way interaction, however did not reach statistical significance, *F* < 1. Planned comparisons were used to further test our *a priori* hypotheses. In line with our expectations, there was a significant linear decrease, *F*(1, 56) = 7.18, *p* < 0.01, in the startle responses with decreasing GS similarity to the CS+ in the predictable condition, but not in the unpredictable condition, (*F* < 1). This generalization gradient was present in the first generalization block, but not in the second generalization block (both *F*s < 1). During the first generalization block, startle responses were still significantly higher for the CS+ than for the CS− in the predictable group, *F*(1, 56) = 6.12, *p* < 0.05, but not in the unpredictable group, (*F* < 1), and this difference was no longer significant in the second generalization block (*F* < 1). Contextual pain-related fear appeared to be attenuated during the first generalization block, ITI startle responses tended to be higher in the unpredictable group compared with the unpredictable group but this difference was no longer statistically significant, *F*(1, 56) = 3.22, *p* = 0.08. During generalization block 2, however, this difference did reach statistical significance, *F*(1, 56) = 6.28, *p* < 0.05. This effect was largely driven by the further decrease in startle responding during the ITI in the predictable group. The generalization gradient was further supported by multiple planned comparisons (see Table 2).

**Table 2:** ***p*-Values for the multiple comparisons using Fisher’s LSD test for the verbal fear ratings and the startle modulation for the predictable group during the first block of the generalization phase**.

	CLASS1 (*p*)	CLASS2 (*p*)	CLASS3 (*p*)	CS− (*p*)
**STARTLE MODULATION**				
CS+	0.443	0.009	0.043	<0.001
CLASS1		0.066	0.208	0.001
CLASS2			0.558	0.166
CLASS3				0.049
**VERBAL FEAR RATINGS**				
CS+	<0.001	<0.001	<0.001	<0.001
CLASS1		0.430	0.641	0.562
CLASS2			0.746	0.834
CLASS3				0.909

### Fear of movement-related pain ratings

#### Fear acquisition

**Figure 4:**
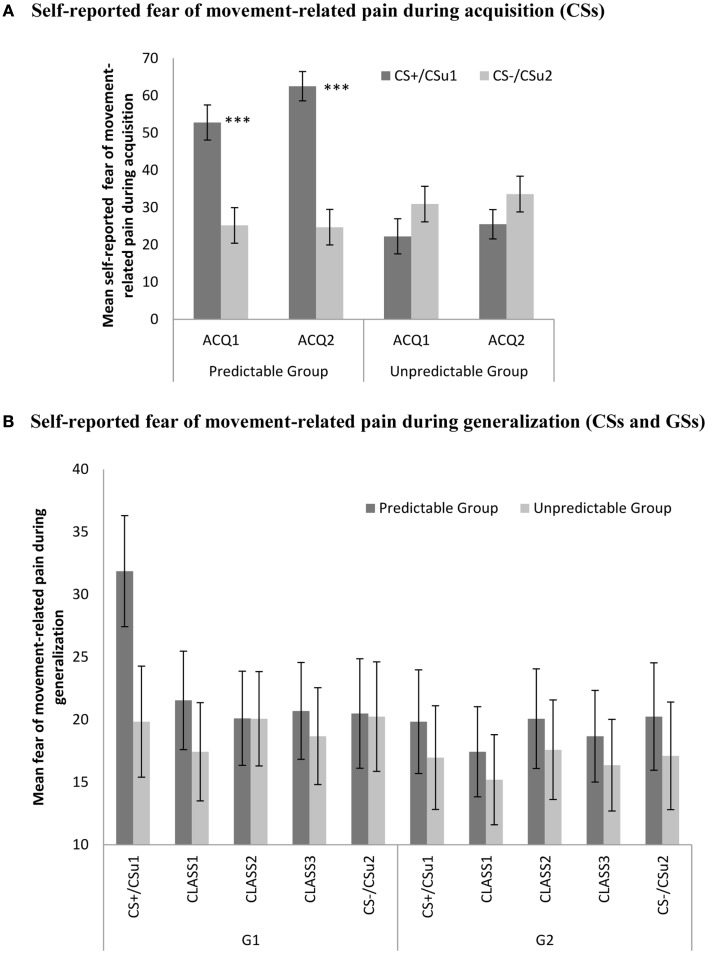
**(A)** Mean self-reported fear of movement-related pain (+SE’s) during the CS movements in the predictable (CS+/CS−) and the unpredictable group (CS_u_1/CS_u_2) during the two acquisition blocks (ACQ1-2), and **(B)** mean eyeblink startle amplitudes (+SE’s) during the CS movements and the classes of GS movements (CLASS1–3) in the predictable and the unpredictable group during the generalization blocks (G1–G2). **(A)** Self-reported fear of movement-related pain during acquisition (CSs). **(B)** Self-reported fear of movement-related pain during generalization (CSs and GSs).

Data were analyzed with a 2 (Group: predictable vs. unpredictable) × 2 (Block: ACQ1 vs. ACQ2) × 2 (Stimulus type: CS+ vs. CS−) ^3^ mixed (RM)ANOVA. This analysis revealed significant main effects for stimulus type, *F*(1, 56) = 21.27, *p* < 0.001, and for group, *F*(1, 56) = 6.58, *p* < 0.05, the latter effect indicating that participants in the predictable group reported higher fear of movement-related pain compared to the unpredictable group. Of crucial importance, the stimulus type × group effect was significant, *F*(1, 56) = 60.71, *p* < 0.001. Planned comparisons at the end of acquisition (ACQ2) confirmed that participants in the predictable group reported significantly more fear of the CS+ compared to the CS−, *F*(1, 56) = 74.91, *p* < 0.001, while participants in the unpredictable group did not give differential fear ratings for both movements, *F*(1, 56) = 10.06, *p* = 0.07 (see Figure 4).

#### Fear generalization

We performed a 2 (Group: predictable vs. unpredictable) × 2 (Block: G1 vs. G2) × 5 (Stimulus type: CS+ vs. class1 vs. class2 vs. class 3 vs. CS−) mixed (RM)ANOVA. The main effect of stimulus type, *F*(4, 224) = 3.10, *p* < 0.05, was significant. There was also a significant main effect of block, *F*(1, 56) = 4.13, *p* < 0.05, indicating that the fear of movement-related pain ratings were declining across blocks. The stimulus type × block interaction effect, *F*(4, 224) = 4.65, *p* < 0.01, was significant, but the anticipated stimulus type × group interaction, *F*(4, 224) = 1.18, *p* = 0.32, and the three-way interaction did not reach statistical significance, *F*(4, 224) = 1.98, *p* = 0.10. Trend analysis further revealed a significant linear, *F*(1, 56) = 8.07, *p* < 0.01, and quadratic, *F*(1, 56) = 8.40, *p* < 0.01, in the predictable condition, but not in the unpredictable condition, [linear: *F* < 1, quadratic: *F*(1, 56) = 1.39, *p* = 0.24]. This trend was present in the first generalization block, but not in the second generalization block (both *F*s < 1). During the first generalization block, fear of movement-related pain ratings were still significantly higher for the CS+ than for the CS− in the predictable group, *F*(1, 56) = 8.20, *p* < 0.01, but not in the unpredictable group, (*F* < 1), and this difference was no longer significant in the second generalization block (*F* < 1). These trends in the beginning of the generalization phase were further examined using multiple planned comparisons (see Table 2).

### Manipulation checks

#### Retrospective pain-US expectancy

We performed a 2 (Group: predicable vs. unpredictable) × 2 (Stimulus type: CS+ vs. CS−) mixed ANOVA on the retrospective pain-US expectancy ratings (see Table 3). This analysis showed a significant main effect for stimulus type, *F*(1, 56) = 11.52, *p* < 0.01, and a significant stimulus type × group interaction, *F*(1, 56) = 62.90, *p* < 0.001, indicating that US expectancy during the respective CS movements was different in both groups. In line with our expectations, participants in the predictable group expected the pain-US to occur more when they performed the CS+ movement than when performing the CS− movement, *F*(1, 56) = 64.13, *p* < 0.001. This difference also turned out to be significant in the unpredictable group, *F*(1, 56) = 10.29, *p* < 0.01, but to a much smaller extent.

**Table 3:** **Manipulation. check measures: mean SAM unhappiness ratings and retrospective pain-US expectancy ratings (and SD) for the CS_p_+ and CS_p_− in the predictable group and the CS_u_1 and CS_u_2 in the unpredictable group**.

*N* = 58	Predictable group		Unpredictable group	
	
	
	CS_p_+	CS_p_−	CS_u_1	CS_u_2
SAM unhappiness (Likert scale; 1–5)	3.17 (0.85)	2.17 (0.71)**	2.55 (0.91)	2.79 (0.77)
Retrospective pain-US expectancy (VAS; 0–100)	68.66 (20.63)	25.45 (21.09)**	36.17 (22.05)	53.48 (24.68)*

*p < 0.01; **p < 0.001; for the CS_p_+/CS_p_− differences and the CS_u_1/CS_u_2 differences in the predictable and the unpredictable group respectively.

#### Retrospective affective valence of the CSs

A 2 (Group: predictable vs. unpredictable) × 2 (Stimulus type: CS+ vs. CS) mixed ANOVA was run on the SAM happiness ratings (see Table 3). There was a significant main effect for stimulus type, *F*(1, 56) = 6.55, *p* < 0.05. More importantly, the stimulus type × group interaction, *F*(1, 56) = 17.55, *p* < 0.001, was significant. To break down this interaction, planned comparisons were calculated. As expected, participants were less happy when performing the CS+ movement compared to the CS− in the predictable group, *F*(1, 56) = 22.77, *p* < 0.001, while there was no such difference in the unpredictable group, *F*(1, 56) = 1.33, *p* = 0.25.

## Discussion

Recent experimental evidence suggests that generalization learning might be involved in the spreading of fear and avoidance behavior that characterizes many musculoskeletal pain disorders (Meulders and Vlaeyen, 2013a). In addition, there is a growing interest for the study of fear generalization and in particular the possible psychological and neurological processes that could influence the process of fear generalization (Vervliet et al., 2010a,b; Lenaert et al., 2012). However, in the field of pain, research on this topic received little attention so far. Therefore, the present study, building on the previous work in our lab, aimed to investigate whether pain predictability by either, a discrete cue (predictable) or contextual cues (unpredictable) affects the degree of generalization learning to novel cues that were never paired with pain. We hypothesized that (1) cued pain-related fear spreads selectively to novel movements that are more similar to the CS+ than to those similar to the CS− (i.e., fear generalization gradient in the predictable group), (2) generalization gradients are flattened in the unpredictable group compared with the predictable group.

*First*, our findings corroborate previous research in establishing *cued pain-related fear* in response to a movement that was consistently paired (CS+) with a pain-US relative to a movement that was never paired (CS−) with pain (Meulders et al., 2011, 2012; Meulders and Vlaeyen, 2012, 2013a,b). This was evident in the eyeblink startle data as well as in the verbal fear ratings in the predictable group. The unpredictable group on the other hand, did not demonstrate such differential conditioned responding for the unreinforced movements in either of the dependent measures. In line with previous research, the ITI startle amplitudes were significantly elevated in the unpredictable group compared with the predictable group, indicating that participants in the unpredictable group acquired *contextual pain-related fear*.

*Second*, we successfully demonstrated a fear generalization gradient in the predictable group, but not in the unpredictable group in both the verbal fear ratings as in the eyeblink startle measures. That is, there was a linear decrease in fear responses for the generalization movements (GSs) approaching the original CS− in both measures. However, this trend was more pronounced in the startle measures than in the verbal fear ratings. Moreover, there was an additional quadratic decrease in fear responses for the generalization movements approaching the original CS− in the startle measures. These data seem to suggest that the linear trend in the verbal ratings is rather driven by the CS+ and CS− differences than by the gradual decline in conditioned responding in response to the GS movement classes based on their similarity with the CSs. This interpretation is further supported by the presence of the quadratic trend in the verbal ratings (which was not present in the startle data). These linear trends were only present in the first generalization block but not in the second. There are several explanations for the absence of the gradient in the second generalization block and the absence of a true gradient in the verbal ratings. The generalization test was performed under extinction, hence CSs and GSs were unreinforced during this phase. Under condition of extinction there might be a steady decline in fearful responding that already starts within a few trials. The verbal ratings were assessed *retrospectively* after each block, that is, after not less than 32 trials, therefore partial extinction probably already took place at the test of the first block. These extinction effects combined with ongoing discrimination learning (differences between the CS+/CS− and the GS movement classes) during the generalization phase might explain the absence of a gradient in the fear ratings. Although the CS+ are still higher than for the CS− and the GS classes during generalization, compared with the end of the acquisition phase, the fear ratings have significantly decreased (mean fear ratings CS+ during ACQ2 = 62.48 vs. mean fear ratings CS+ during G1 = 31.86 vs. mean fear ratings CS+ during G2 = 19.83) which supports our *post hoc* interpretation of combined extinction and ongoing discrimination learning during the unreinforced generalization phase. This is not the case for the startle measures, because probes are delivered throughout the generalization test providing a more online representation of the fear responses. In line with our *post hoc* explanation, the difference in fear reported to the CS+ and the CS− declined significantly from the end of acquisition to the first generalization block, *F*(1, 56) = 23.22, *p* < 0.001, whereas this decline was not the significant in the startle measures *F*(1, 56) = 1.33, *p* = 0.25. Follow-up studies might consider using a partial reinforcement scheme during generalization testing.

*Third*, some procedural aspects deserve some more attention. For instance, another design feature that was different compared with the previous study by Meulders and Vlaeyen (2013a) is that the trial timing is more spread out as a BSs design was used instead of a WSs design [meaning that the relative (un)predictability of the US could not be derived from the experience with both conditions]. These features might have influenced the safety learning in the unpredictable group. That is, verbal fear ratings in response to the unreinforced movements in the unpredictable group are much lower than typically observed using this paradigm so far. Probably, because delayed trace conditioning is less likely to occur when inter intervals are longer and thus spilling-over of contextual fear to the technically safe movements is less obvious, suggesting that the unreinforced movements in the unpredictable group truly gained inhibitory properties.

Some limitations are worthy to discuss as well. *First*, the movements that the participants had to perform were not chosen voluntarily, like previous studies using a similar paradigm (Meulders et al., 2011, 2012; Meulders and Vlaeyen, 2012, 2013a), but instead were signaled by a number corresponding with the quadrant in which they had to move presented before each movement. This was mainly done for statistical purposes, as it assured an equal number of startle probes delivered during each of the movements. Nevertheless, one could argue that the number cue, and not the movement *per se* acted as a predictor for the pain-US because there was also a perfect contingency between the number cue and the occurrence of the pain-US. This is rather unlikely for at least two reasons: (1) because it is not just an inert cue, the movement is probably more salient which in turn leads to an advance in terms of cue competition (De Houwer and Beckers, 2002; Shanks, 2010), and (2), following the componential CS representation view (Vogel et al., 2003), late components of a CS typically gain excitatory properties by virtue of their temporal proximity with the US, whereas early components of a CS gain inhibitory properties. In the present study, the complex CS might comprise an early visual component (number cue) and a late proprioceptive component (movement). Hence, it would be predicted that participants would learn that the pain-US will not occur (i.e., inhibitory learning) before they actually performed the movement. *Second*, during the practice phase, the participants were already pre-exposed to the generalization stimuli which might have attenuated the generalization gradient. Generalization is the opposite of discrimination, which is the degree to which the cortical representation of one stimulus can be distinguished from that of other stimuli. In other words, the precision by which the representation of a stimulus is encoded is negatively related to the array of stimuli that it can activate (Flor et al., 2001). Therefore, if participants are familiarized with the GSs before the generalization test, it is possible that differences with the CSs are encoded in more detail leading to weakened generalization. *Third*, we only included female participants in our study so the results cannot be generalized to a male population. There are some indications that woman react with stronger pain sensitization to (un)predictable situations than men do (Meulders et al., 2012).

In conclusion, this study provided further evidence for the notion that associative learning is involved in the acquisition of cued and contextual pain-related fear. More importantly, this is the first study to demonstrate a generalization gradient of cued pain-related fear (predictable pain group), that is, more spreading of fear toward novel movements that are proprioceptively related to the original painful movement than to the ones resembling the non-painful movement. There was no such generalization gradient for contextual pain-related fear (unpredictable pain group). Taken together, this paradigm represents a novel tool to scrutinize the largely understudied phenomenon of fear generalization in patients with chronic musculoskeletal pain and mapping possible pathological differences in generalization gradients and the spreading of pain in patients as compared with healthy controls. Future research might focus on the conditions under which these generalization gradients can be broadened or reduced in order to develop new methods to limit the spreading of fear of movement in chronic pain patients. This line of research warrants further investigation, especially given the intriguing but untested idea that based on the close relationship between fear and pain, fear generalization might be associated with subsequent spreading of pain.

## Conflict of Interest Statement

The authors declare that the research was conducted in the absence of any commercial or financial relationships that could be construed as a potential conflict of interest.

## Footnotes

^1^Note that the term movement area has a different meaning than target area in this context, the latter being defined as the region that participants had to reach in order to change the color of the blue bars, whereas the former refers to the area within which they move the joystick before reaching a given target region.

^2^The WS factor stimulus type had one extra level in the startle modulation analysis, i.e., the ITI representing the contextual fear measure. In particular, during acquisition this WS factor included two levels (CS+/CS−) for the verbal ratings and three levels (CS+/CS−/ITI) for the startle modulation. During generalization this WS factor included five levels (CS+/CLASS1/CLASS2/CLASS3/CS−) for the verbal ratings and six levels (CS+/CLASS1/CLASS2/CLASS3/CS−/ITI) for the startle modulation.

^3^Note that throughout this paper, the notations CS+ and CS−used in the descriptions of the statistical analyses and the figures, respectively refer to the CS_p_+ and the CS_p_−in the predictable group, and to the unreinforced CSs, (i.e., CS_u_1 and CS_u_2 in the unpredictable context).
